# Corosolic Acid Attenuates the Invasiveness of Glioblastoma Cells by Promoting CHIP-Mediated AXL Degradation and Inhibiting GAS6/AXL/JAK Axis

**DOI:** 10.3390/cells10112919

**Published:** 2021-10-28

**Authors:** Li-Wei Sun, Shao-Hsuan Kao, Shun-Fa Yang, Shang-Wun Jhang, Yi-Chen Lin, Chien-Min Chen, Yi-Hsien Hsieh

**Affiliations:** 1 Institute of Medicine, Chung Shan Medical University, Taichung 40201, Taiwan; medicaldragon007@gmail.com (L.-W.S.); kaosh@csmu.edu.tw (S.-H.K.); ysf@csmu.edu.tw (S.-F.Y.); mo915915@gmail.com (Y.-C.L.); 2 Division of Neurosurgery, Department of Surgery, Changhua Christian Hospital, Changhua 50006, Taiwan; 133393@cch.org.tw; 3 Department of Medical Research, Chung Shan Medical University Hospital, Taichung 40201, Taiwan; 4 Department of Veterinary Medicine, National Chung Hsing University, Taichung 40201, Taiwan; 5 School of Medicine, Kaohsiung Medical University, Kaohsiung 80708, Taiwan; 6 College of Nursing and Health Sciences, Dayeh University, Changhua 51591, Taiwan

**Keywords:** corosolic acid, glioblastoma cell, invasiveness, AXL, CHIP, GAS6, JAK2

## Abstract

Corosolic acid (CA), a bioactive compound obtained from Actinidia chinensis, has potential anti-cancer activities. Glioblastoma (GBM) is a malignant brain tumor and whether CA exerts anti-cancer activity on GBM remains unclear. This study was aimed to explore the anticancer activity and its underlying mechanism of CA in GBM cells. Our findings showed that CA ≤ 20 μM did not affect cell viability and cell proliferative rate of normal astrocyte and four GBM cells. Notably, 10 or 20 μM CA significantly inhibited cell migration and invasion of three GBM cells, decreased the protein level of F-actin and disrupted F-actin polymerization in these GBM cells. Further investigation revealed that CA decreased AXL level by promoting ubiquitin-mediated proteasome degradation and upregulating the carboxyl terminus of Hsc70-interacting protein (CHIP), an inducer of AXL polyubiquitination. CHIP knock-down restored the CA-reduced AXL and invasiveness of GBM cells. Additionally, we observed that CA-reduced Growth arrest-specific protein 6 (GAS6) and inhibited JAK2/MEK/ERK activation, and GAS6 pre-treatment restored attenuated JAK2/MEK/ERK activation and invasiveness of GBM cells. Furthermore, molecular docking analysis revealed that CA might bind to GAS6 and AXL. These findings collectively indicate that CA attenuates the invasiveness of GBM cells, attributing to CHIP upregulation and binding to GAS6 and AXL and subsequently promoting AXL degradation and downregulating GAS6-mediated JAK2/MEK/ERK cascade. Conclusively, this suggests that CA has potential anti-metastatic activity on GBM cells by targeting the CHIP/GAS6/AXL axis.

## 1. Introduction

Glioma is the most common form of brain tumor, and glioblastoma (GBM) is the most malignant glioma, accounting for 3–4% of all cancer-associated deaths [1]. The five-year survival rate for patients with GBM is approximately 4–5%, indicating that the prognosis of GBM is poor [2]. The standard treatment for GBM includes resection with concurrent radiotherapy and chemotherapy. However, the current standard treatment did not significantly increase the survival rate of patients with GBM compared with those with glioma and other subtypes [3]. Due to the introduction of alkylating agents, such as temozolomide and adjuvant therapy combined with radiotherapy and temozolomide, the median survival time of patients with GBM increased from 12.1 months to 14.6 months. [4,5]. However, the inherent or induced resistance to temozolomide leads to unsatisfactory clinical efficacy of GBM. Therefore, new therapies for this deadly tumor still need a more comprehensive understanding of its progress, drug resistance mechanisms and novel therapeutic targets.

Abnormal activation of receptor tyrosine kinase (RTK) is highly correlated with tumorigenesis, leading to uncontrolled proliferation, inhibition of apoptosis, and promotion of metastasis. Among RTK family, the TAM (Tyro-3, AXL, Mer) kinases have been implicated in the development of a serial of cancers [6,7]. TAM kinases are overexpressed in numerous cancers, including myeloid and lymphoblastic leukaemia, breast, lung, colon, liver, gastric, kidney and brain cancers [8,9,10]; particularly, both overexpression of AXL and its ligand growth arrest specific 6 (Gas6) have been reported as poor prognosis markers in GBM patients [11]. Downstream signaling of AXL and Mer results in a serial oncogenic mechanism including cell growth and survival, metastasis, angiogenesis, and chemoresistance in solid tumors [12]. In addition, AXL also plays an important role in regulation of glioblastoma stem-like cells [13]. Therefore, it is suggested that inhibition of AXL and GAS6 could be a promising target for GBM treament [14].

Corosolic acid (CA) is a pentacyclic triterpene compound that can be extracted from the leaves of *Eriobotrta japonica* [15], the fruit of *Cratoegus pinnatifida* var. psilosa [16], and the root of *Actinidia chinensis* [17]. Recently, the anti-tumor activity of CA has attracted more attention [18,19]. CA possesses cytotoxic activity to cervical cancer [20], hepatocellular carcinoma [17], and lung cancer [19]. Fujiwara et al. also reported that CA could inhibit proliferation of glioblastoma cell and M2 polarization of tumor-associated macrophages (TAMs) [18]; however, whether CA has anti-metastatic activity on GBM cells is incompletely studied. Therefore, in this study, anti-metastatic potential of CA on GBM cells is first explored with emphasis on AXL and its associated signal components.

## 2. Materials and Methods

### 2.1. Reagents and Antibodies

Chemicals and reagents were obtained from Sigma-Aldrich (St. Louis, MO, USA) or as indicated. Corosolic acid (CA; Purity ≥ 98%) was purchased from ChemFaces company (Wuhan, Hubei, China). The antibodies source and dilution factor were: F-actin (200 µg/mL; dilution factor: 1:1000), AXL (200 µg/mL; dilution factor: 1:1000), Ubiquitin (200 µg/mL; dilution factor: 1:1000), CHIP (200 µg/mL; dilution factor: 1:1000), GAS6 (200 µg/mL; dilution factor: 1:1000), phospho(p)-JAK2 (200 µg/mL; dilution factor: 1:1000), pan-JAK (200 µg/mL; dilution factor: 1:1000), p-MEK-1/2 (Ser 218/Ser222; 200 µg/mL; dilution factor: 1:1000), pan-ERK (200 µg/mL; dilution factor: 1:1000), p-MEK (200 µg/mL; dilution factor: 1:1000), pan-MEK (200 µg/mL; dilution factor: 1:1000), GAPDH (200 µg/mL; dilution factor: 1:5000), and peroxidase-conjugated antibodies against mouse IgG (200 µg/mL; dilution factor: 1:5000) or rabbit IgG (200 µg/mL; dilution factor: 1:5000) were obtained from Santa Cruz Biotechnology (Santa Cruz, CA, USA). The phospho(p)-ERK1/2(Thr202/Tyr204), (dilution factor: 1:1000), GAS6 (dilution factor: 1:1000), Phospho-JAK2 (Tyr1007/1008), (dilution factor: 1:1000) were purchase from Cell Signaling Technology (Beverly, MA, USA). MG132 and Cycloheximide were purchased from Selleck Chemicals (Houston, TX, USA). Recombinant Human GAS6 Protein (Rh-GAS6) was purchased from R&D Systems, Inc (Minneapolis, MN, USA)

### 2.2. CA Treatment

The stock concentration of CA is 50 mM and dissolved in dimethyl sulfoxide (DMSO) at −20 °C and diluted using the culture medium with a final DMSO concentration of 0.1%. MTT assay were detected cell viability and cytotoxicity by using the CA concentration at 10, 15, 20, 25 and 30 μM for 24 and 48 h. The CA concentrations used in colony formation, cell cycle, apoptosis, in vitro migration/invasion assay and western blotting at 10, 15 and 20 μM for 24 h. Control were treated with same amount of DMSO as corresponding group in this study.

### 2.3. Cell Culture

Rat astrocyte CTX-TNA2 cells was established from primary cultures of astrocytes in old rats (brain frontal cortex tissue) and kindly provided from Dr. Nu-Man Tsai (School of Medical Laboratory and Biotechnology, Chung Shan Medical University, Taichung, Taiwan). The U251-MG cell lines were a gift from Professor Dah-Yu Lu of China Medical University (Taichung, Taiwan). The astrocyte CTX-TNA2 cells was maintained in Dulbecco’s Modified Eagle’s Medium (DMEM) with 4.5 g/L glucose and 10% (*v*/*v*) fetal bovine serum (FBS). GBM8401, M059K and U-87MG were acquired from BCRC (Bioresources Collection and Research Center, Hsinchu, Taiwan). Additionally, cells were grown in Dulbecco’s modified Eagle’s medium (DMEM) supplemented with 10% (*v*/*v*) FBS and 1% penicillin/streptomycin at 37 °C in a humidified CO_2_ (5%)-controlled incubators. Finally, subculture was performed when cells reached 80% confluency.

### 2.4. Cell Viability Assay

Cell viability was determined using Thiazolyl Blue Tetrazolium Bromide (MTT) assay as previously described [21]. Briefly, 2 × 10^4^ cells were seeded into a 24-well plate and treated with CA at 10, 15, 20, 25, and 30 µM for 24 or 48 h (h), and then incubated with the MTT solution. After adding isopropanol to solubilize the formed formazan, the absorbance of the solution at 563 nm was measured using a spectrophotometer. The percentage of viable cells was estimated by comparing with control.

### 2.5. Colony Formation Assay

Cells (4 × 10^5^) were seeded onto 6-well culture plates and then incubated with treated with CA at 10, 15 and 20 μM and then incubated at 37 °C for 7 days. At the end of incubation, the cell colonies were fixed with methanol, stained with crystal violet (1:20), then photographed using a light microscopy. The colonies were counted for quantitation by ImageJ software.

### 2.6. Migration and Invasion Ability by Boyden Chamber Assay

First, cells were incubated in serum-free DMEM containing CA at 10, 15 and 20 μM and then seeded on 24-well cell culture inserts using 8 μm GVS PCTE Filter Membranes (GVS Life Sciences, Zola Predosa, Bologna, Italy). Next, 20% FBS was added to the lower chamber and used as the chemoattractant. After 24 h of incubation, cells that migrated to the lower surface of the insert were fixed with 10% neutral-buffered formalin and stained with Giemsa reagent (Millipore). Then, the stained cells were photographed, and the total cell number from five random fields was counted by light microscopy. For the invasion assay, 100-μL Matrigel (20× dilution in PBS) was added to the culture inserts and then air-dried before cell seeding (as described above).

### 2.7. Immunofluorescence Staining

Cells were fixed by 4% ice-cold formaldehyde, reacted with blocking buffer containing 5% bovine serum albumin (BSA) and 0.5% Triton X-100 in PBS solution for 1 h at 25 °C, and incubated with primary antibodies for 16 h at 4 °C. Next, the cells were washed with PBS, and the bound primary antibodies were detected using F-Actin Labeling Kit; Red Fluorescence (AAT Bioquest, Inc., Sunnyvale, CA, USA) was used to detect polymerized F-actin microfilaments. Finally, fluorescence images were acquired using a laser scanning confocal microscope (Zeiss 510-Meta, Zeiss, Oberkochen, Germany).

### 2.8. Western Blot

Western blot was conducted as previously described [22]. Briefly, cells were lysed in Tris lysis buffer containing protease and phosphatase inhibitor cocktail (Sigma-Aldrich). Then, the resulting crude proteins were separated by sodium dodecyl sulphate-polyacrylamide gel electrophoresis (SDS-PAGE), transferred to Immobilon-P polyvinylidene difluoride (PVDF) membrane (Merck, Kenilworth, NJ, USA) and then reacted with primary antibodies followed by secondary antibodies. The bound antibodies were detected using Immobilon Western Chemiluminescent HRP Substrate (Merck, Darmstadt, Germany) and an image analysis system by LAS-4000 mini (GE Healthcare Bio-Sciences, Piscataway, NJ, USA). Densitometric analysis was performed for semi-quantitation of chemiluminescence signals.

### 2.9. Quantitative Real-Time Polymerase Chain Reaction (qPCR)

After treatment, cells were harvested and then lysed for total RNA extraction using Isol-RNA-Lysis Reagent (Gaithersburg, MD, USA). The complementary DNA (cDNA) was ac-quired by reverse transcription of total RNA using the ReverTra Ace qPCR RT Master Mix kit (TOYOBO, Osaka, Japan). Then, qPCR was conducted using a StepOne Real-Time PCR System (Applied Biosystems, Foster City, CA, USA). The primers used for human gene expression by qPCR included AXL, (F) 5′-GTT TGG AGC TGT GAT GGA AGG C-3′, (R) 5′-CGC TTC ACT CAG GAA ATC CTC C-3′ (NM_021913, OriGene, Mission Biotech, Taipei, Taiwan). Finally, relative gene expression quantitation was normalized with endogenous GAPDH using the 2^−ΔΔCt^ method.

### 2.10. Knockdown of CHIP by Small Inhibitory RNAs

CHIP expression knockdown was conducted using specific small inhibitory RNAs (siR-NAs) according to the manufacturer’s protocol. Briefly, GBM8401 cells were transfected with CHIP siRNA into a pool of three siRNA duplexes (si-CHIP; sc-43555A, sc-43555B and sc-43555C) and a scrambled control siRNA (Santa Cruz Biotechnology, CA, USA). The siRNA transfection reagent used was Lipofectamine RNAiMAX (Thermo Fisher Scientific Inc., Waltham, MA, USA) at 37 °C and 5% CO_2_ for 72 h.

### 2.11. Molecular Docking Approach

Binding mode and selectivity of AXL kinase and GAS6 with CA were studied using AutoDock Vina [23], which required the ligand (GAS6: 1H30) and receptor (AXL: 5U6B) in RCSB protein database bank (PDB, http://www.rcsb.org; GAS6: accessed on 30 January 2003; AXL: accessed on 26 July 2017). Additionally, CAs structure was downloaded from NCBI PubChem (CID: 6918774). Molecular docking score was calculated using mcule with Autodock vina. The program PyMOL (http://www.pymol.org/; GAS6: accessed on 15 December 2009; AXL: accessed on 15 December 2009) was analyzed for visualizing 3D structures.

### 2.12. Statistical Analysis

The data from three independent experiments were presented as the mean ± standard deviation (SD) except indicated. Student’s *t*-test and one-way analysis of variance (ANOVA) followed by Dunnett’s post hoc test were used to analyze significant differences, and results with *p* < 0.05 or *p* < 0.01 were considered statistically significant.

## 3. Results

### 3.1. Effects of CA on the Cell Viability and Colony Formation Potential of Normal Astrocyte and GBM Cells

**Figure 1:**
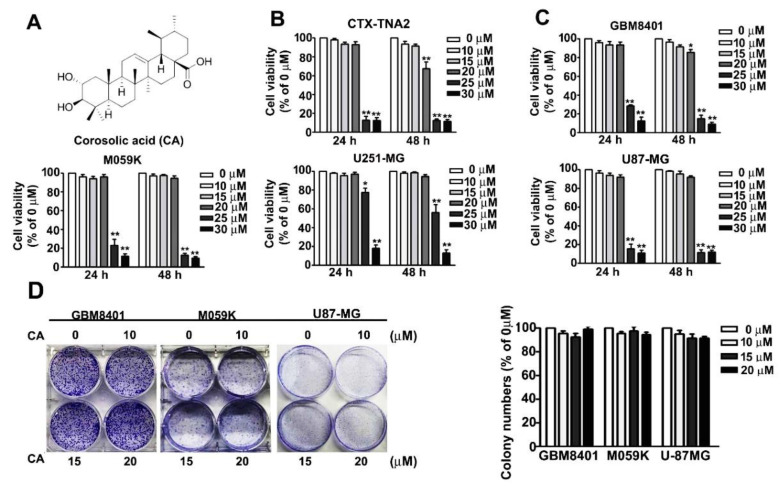
Effect of CA on cell viability and colony formation of GBM cells. (**A**) Structure of CA. (**B**,**C**) Normal astrocyte, CTX-TNA2 and GBM cell lines, GBM8401, M059K, U251-MG and U87-MG, were treated with CA at the indicated concentrations for 24 or 48 h. Then, cell viability was assessed by MTT assay and presented as a percentage of control. (**D**) GBM8401, M059K and U87-MG were seeded onto cell culture dishes containing without or with CA at 10, 15 and 20 μM for 7 days, and then cell colonies were stained with Giemsa and counted using a light micro-scope. Three independent experiments were performed for statistical analysis. * *p* < 0.05; ** *p* < 0.01 compared with control (DMSO-treated cells).

CA’s structure is shown in Figure 1A, and its effects on cell viability of normal astrocytes, CTX-TNA2 and human GBM cell lines, GBM8401, M059K, U251-MG, and U87-MG, were first explored. After 24- or 48-h treatments, cell viability was remarkably reduced by CA at 25 and 30 μM (*p* < 0.05), but unaffected by CA at 10, 15 and 20 μM compared with the control (Figure 1B,C). Notably, an exception showed that 20 μM CA treatment for 48 h could decrease the cell viability of CTX-TNA2 cells to 84.7% ± 5.3% of control (*p* < 0.05) were detected by MTT assay. Then, we evaluated the effects of low-dose CA (10, 15 and 20 μM) on the colony formation potential of GBM cells. Our results showed that low-dose CA treatment did not influence the colony formation potential of GBM8401 cells (Figure 1D). Therefore, CA at 10, 15 and 20 μM were used for further cell experiments.

### 3.2. Effects of CA on the Cell Cycle and Cell Death of Three GBM Cells

**Figure 2:**
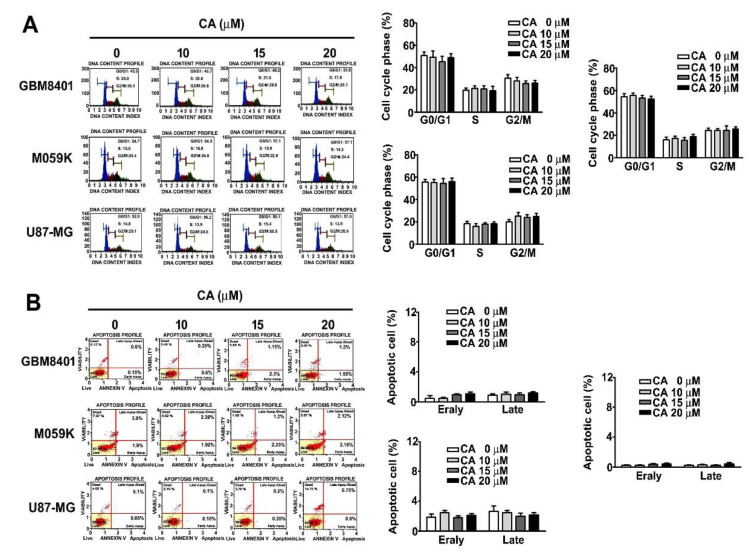
Effect of CA on cell cycle and death of GBM cells. (**A**) GBM cell lines, GBM8401, M059K, U251-MG and U87-MG, were treated with CA at the indicated concentrations for 24 h, and then the cell cycle was detected with PI staining assay by flow cytometry. (**B**) Cell death was measured by Annexin V/PI staining using a flow cytometer and presented as a percentage of the control.

Low-dose CA treatments do not affect the cell viability and proliferation ability of GBM cells. To determine which cell cycle arrest or cell death on GBM8401, M059K and U-87MG cells was influenced by CA. Our results showed that no effect on cell cycle distribution (G0/G1, S or G2/M phase) in CA-treated GBM8401, M059K and U87-MG cells, which was shown by PI (propidium iodide) staining using a flow cytometer (Figure 2A). However, we also observed that CA does not affect cell death of GBM8401, M059K and U87-MG by Annexin V/PI staining assay (Figure 2B). These pieces of evidence suggest that low-dose CA treatment is independent on cell viability and death.

### 3.3. CA Attenuates the Invasiveness of Human GBM Cells and Reduces F-Actin Expression

**Figure 3:**
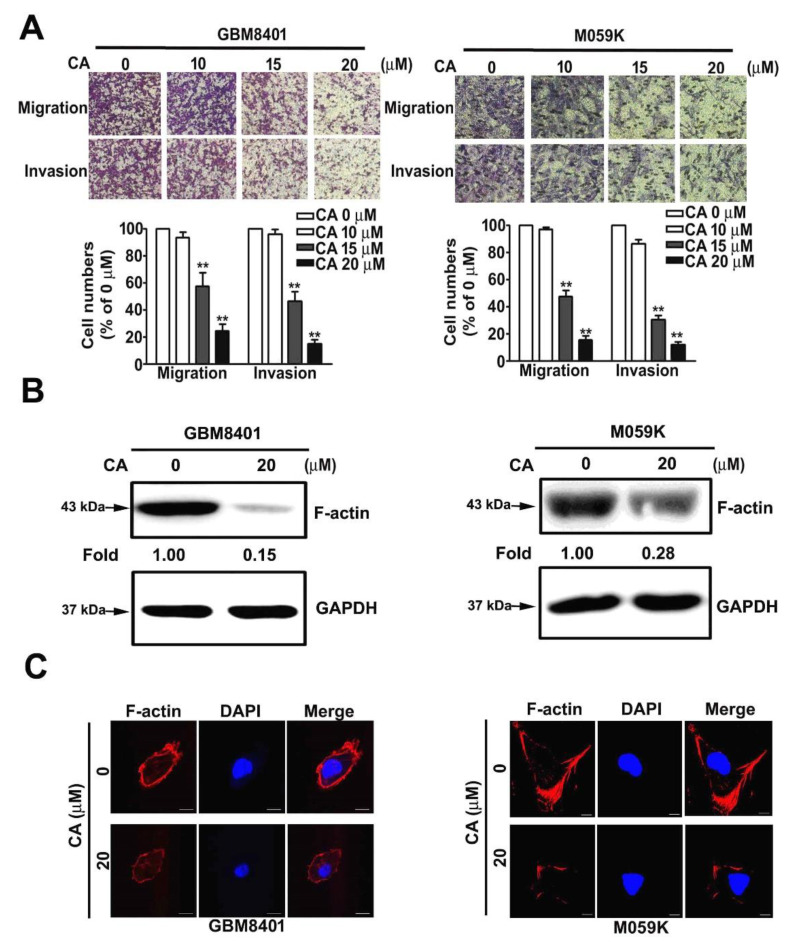
CA attenuated the migration and invasion of GBM cells and reduced expression and organization of cytoskeletal F-actin in GBM cells. (**A**) GBM8401 and M059K cells were treated with CA at indicated concentrations, and then cell migration and invasion were assessed and quantitated as a percentage of the control. ** *p* < 0.01 compared with the control. (**B**) GBM8401 and M059K cells were treated with CA (20 μM) and then lysed for immunodetection of F-actin by Western blotting. GAPDH was used as an internal control. (**C**) GBM8401 and M059K cells were treated with CA (20 μM) and then stained with phalloidin for F-actin (red) and DAPI for the nucleus (blue). Images were acquired using a confocal microscope at 200× magnification. ** *p* < 0.01 compared with the control (DMSO-treated cells). Scale bar = 50 μm.

Since low-dose CA treatment insignificantly affected cell viability and colony formation capability of GBM cells, whether low-dose CA exhibited anti-metastatic activity on GBM cells was further evaluated. CA treatments dose-dependently and significantly attenuated the migratory and invasive potentials of GBM8401 and M059K cells up to 17.5% ± 2.4% and 11.6% ± 1.7% of control, respectively (for 20 μM CA, *p* < 0.01 compared with control at 0 μM; Figure 3A). Furthermore, aberrant regulation of the actin cytoskeleton is highly associated with the invasiveness of tumor cells [24]. Thus, whether CA altered F-actin expression in GBM cells, important cytoskeletal actin involved in tumor metastasis [25], was examined. Our observation showed that 20 μM CA decreased the protein level of F-actin by 0.15- and 0.28-fold of the control in GBM8401 and M059K cells, respectively (Figure 3B). Additionally, 20 μM CA disrupted the F-actin cytoskeletal organization in the three GBM cells (Figure 3C). Collectively, these findings reveal that CA inhibits the invasiveness of GBM cells, downregulates F-actin expression and disrupts the cytoskeletal organization.

### 3.4. CA Reduces the Protein Level of AXL by Promoting Ubiquitin-Mediated Proteasome Degradation

**Figure 4:**
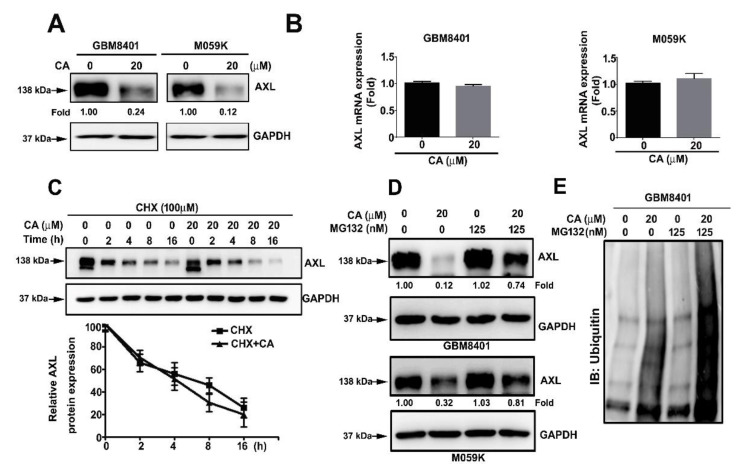
CA decreased AXL protein level by promoting ubiquitin-mediated proteasome degradation in GBM8401 cells (**A**,**B**) GBM8401 and M059K cells were treated with CA at 20 μM for 24 or 6 h, and then lysed for AXL immunodetection by Western blotting (**A**) or for mRNA expression assessment of AXL by RT-qPCR (**B**). (**C**) GBM801 cells were treated with cycloheximide (CHX) alone or with CHX and CA for the indicated times and then lysed for AXL immunodetection by Western blotting. Chemiluminescence signal was semi-quantitated by densitometric analysis, and GAPDH was used as an internal control. (**D**,**E**) GBM8401 and M059K cells were treated with CA, MG132 or CA and MG132 for 24 h and lysed for AXL immunodetection (**D**) or ubiquitin (**E**) by Western blotting.

Next, the mechanism by which CA disrupted the F-actin cytoskeletal organization was investigated. Among the essential cytoskeleton regulators, AXL overexpression, a receptor, tyrosine, has been observed in different cancers and associated with an aggressive phenotype, invasiveness and progression [8,26]. Thus, CA influence on AXL was assessed. In addition, CA treatment decreased the protein level of AXL in GBM8401 and M059K (Figure 4A). Interestingly, CA treatment did not alter the mRNA expression of AXL in both cells (Figure 4B, *p* > 0.05). As a result, whether CA affected the protein stability of AXL was then examined. Compared with inhibition of protein synthesis by cycloheximide (CHX) alone, CA combined with CHX treatments showed an insignificant effect on the stability of AXL protein in GBM8401 cells (Figure 4C). Notably, compared with CA treatment alone, pre-treatment with the proteasome inhibitor, MG132, with CA treatments significantly restored AXL protein levels in GBM8401 and M059K cells (Figure 4D). Moreover, combining MG132 pre-treatment and CA treatment also increased the level of polyubiquitinated proteins in both cells compared with CA treatment alone (Figure 4E). Collectively, these observations indicate that CA downregulates AXL protein levels by promoting ubiquitin-mediated proteasome degradation.

### 3.5. Involvement of CHIP in CA-Reduced AXL and F-Actin and CA-Attenuated Invasiveness of GBM8401 Cell

**Figure 5:**
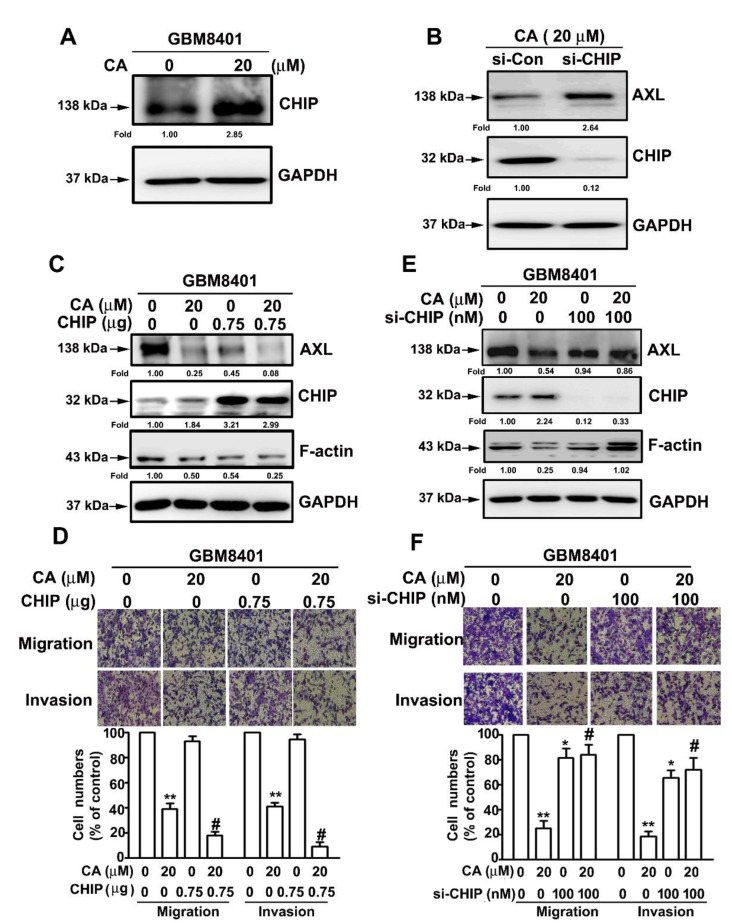
CHIP involvement in CA-inhibited migration and invasion of GBM8401 cells. (**A**,**B**) Cells were treated with CA (20 μM) (**A**) or siRNA against CHIP (si-CHIP) (**B**) and then lysed for CHIP and AXL immunodetection by Western blotting. (**C**,**D**) Cells were treated with CA (20 μM), CHIP (0.75 μg) or CA and CHIP, and then lysed for AXL, CHIP and F-actin immunodetection by Western blotting (**C**) or subjected to migration and invasion assay (**D**). (**E**,**F**) Cells were treated with CA (20 μM), si-CHIP (100 nM) or CA and si-CHIP, and then lysed for AXL, CHIP and F-actin immunodetection by Western blotting (**E**) or subjected to migration and invasion assay (**F**) Chemiluminescence signal was semi-quantitated by densitometric analysis, and GAPDH was used as an internal control. * and ** *p* < 0.05 and 0.01, respectively, compared with the control (DMSO-treated cells). # *p* < 0.05 compared with CA alone. Images were acquired by light microscopy at 200× magnification.

Previous studies indicate that ubiquitin E3 ligase carboxyl terminus of HSC70-interacting protein (CHIP) plays a vital role in AXL degradation [27]. Thus, CHIP involvement in AXL and F-actin downregulation in response to CA was explored. First, CA treatment increased the CHIP protein level in GBM8401 cells (Figure 5A). Then, a specific siRNA against CHIP (si-CHIP) was designed to silence the gene expression of CHIP; results showed that CHIP silencing markedly decreased CHIP protein levels and increased AXL protein levels in GBM8401 cells (Figure 5B). Additionally, CHIP treatment decreased AXL and F-actin levels and increased CHIP levels in GBM8401 cells; CHIP and CA co-treatment further decreased AXL and F-actin levels compared with CHIP and CA alone (Figure 5C). Next, using si-CHIP, we observed that the CA-downregulated AXL and F-actin protein levels were markedly reversed in GBM8401 cells (Figure 5D). Thus, consistent with CHIP changes, CHIP treatment synergistically promoted the inhibitory effects of CA on the migration and invasion of GBM8401 cells (Figure 5E); and silencing CHIP reversed the inhibitory effects of CA on the migration and invasion of GBM8401 cells (Figure 5F). Altogether, these findings reveal that CHIP is involved in AXL and F-actin downregulation induced by CA and the suppression of GBM8401 cell migration and invasion by CA treatment.

### 3.6. Involvement of GAS6 in CA-Attenuated Invasiveness of GBM Cells

**Figure 6:**
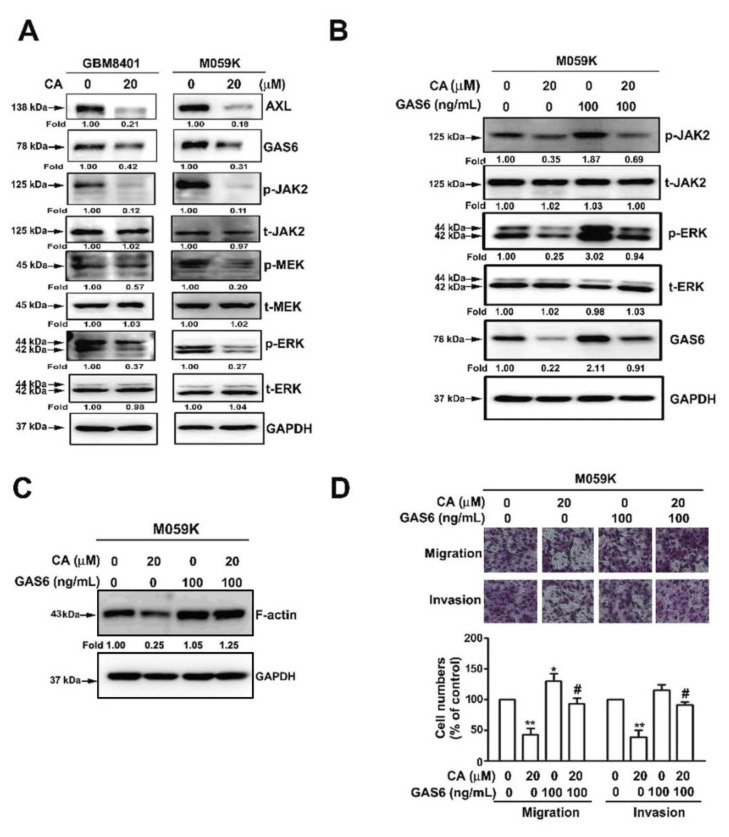
Involvement of GAS6-associated cascade in CA-inhibited migration and invasion of GBM cells. (**A**) Cells were treated with CA (20 μM) and then lysed for immunodetection of the indicated targets by Western blotting. (**B**,**C**) Cells were treated with CA (20 μM), GAS6 (100 ng/mL), or a combination of CA and GAS6, and then lysed for immunodetection of the indicated targets (**B**) or F-actin (**C**) by Western blotting. (**D**) Cells were treated with CA (20 μM), GAS6 (100 ng/mL) or a combination of CA and GAS6, then subjected to migration and invasion assay. Chemiluminescence signal was semi-quantitated by densitometric analysis, and GAPDH was used as an internal control. * and ** *p* < 0.05 and 0.01, respectively, compared with the control (DMSO-treated cells). # *p* < 0.05 compared with CA alone. Images were acquired using a light microscope at 200× magnification.

AXL is also activated by GAS6 (growth arrest-specific 6), a member of vitamin K-dependent proteins [8]. As a result, whether CA affected GAS6 and its associated signaling was then investigated. CA (20 μM) reduced AXL and GAS6 levels in GBM8401 and M059K cells and inhibited JAK2, MEK and ERK phosphorylation in both cells (Figure 6A). With exposure to GAS6, JAK2 and ERK phosphorylation and GAS6 level were increased in M059K cells compared with the control (Figure 6B), and CA diminished the GAS6-induced phosphorylation of JAK2 and ERK and GAS6 level in M059K cells (Figure 6B). Moreover, CA also decreased GAS6-induced F-actin level in M059K cells compared with the GAS6 treatment alone (Figure 6C). By migration and invasion assays, GAS6 treatments promoted the migration and invasion of M059K cells compared with the control (Figure 6D, *p* < 0.05). Notably, CA significantly lowered the migration and invasion of M059K cells exposed to GAS6 than those exposed to GAS6 alone (Figure 6D, *p* < 0.05). Thus, these findings reveal that CA downregulates GAS6 expression level and inhibits GAS6-associated signaling, consequently suppressing the migration and invasion of GBM cells

### 3.7. Docking Study of CA with AXL and GAS6

**Figure 7:**
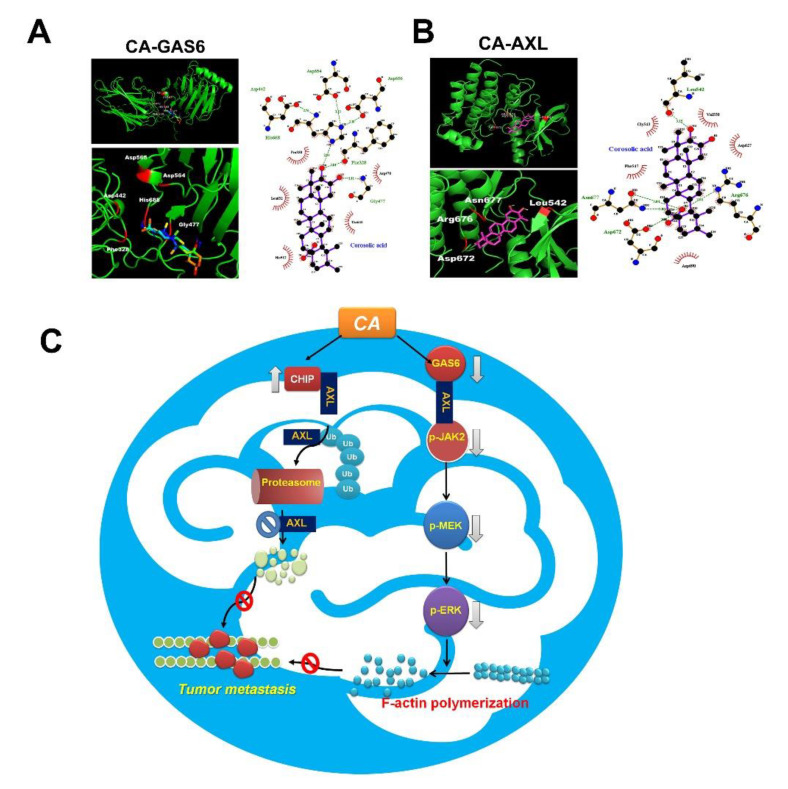
Molecular docking analysis and proposed mechanism for CA-inhibited invasiveness of GBM cells. (**A**) Superposition of GAS6 (green) and template CA (cyan), hydro-gen bonding interactions with GAS6 and CA. Binding affinity: −7.6 kcal/mol. (**B**) Superposition of AXL (green) and template CA (cyan), hydrogen bonding interactions with AXL and CA. Binding affinity: −5.6 kcal/mol. Interacting amino acid residues: Asn677, Arg676, Leu542 and Asp672. (**C**) Proposed mechanism for CA-inhibited invasiveness of GBM cells.

Based on the inhibitory effects of CA on GAS6 and AXL, the possible interaction between CA and GAS6 was investigated by molecular docking. Docking analysis revealed hydro-gen bonds between the Phe328 and His668 of GAS6 and the 10-hydroxy groups of CA and between the Gly477 of GAS6 and 11-hydroxy groups of CA (Figure 7A). Additionally, docking analysis showed hydrogen bonding networks between the Leu542 of AXL and the 10-hydroxy groups of CA and between the Asn677, Arg676 and Asp672 of AXL and the 4a-carboxylic group of CA (Figure 7B). These observations showed that CA exhibited strong binding to GAS6 and AXL, mainly by hydrogen bonding and hydrophobic interactions, which may result in increased CHIP and decreased GAS6, and the consequent promotion of AXL degradation and inhibition of JAK2/MEK/ERK cascade (Figure 7C).

## 4. Discussion

Recently, inhibition of AXL tyrosine kinases has become an important method for cancer treatment. However, most small molecules with an inhibitory activity on AXL kinase are not primarily synthesized for AXL; therefore, the inhibitory activity against AXL is not as robust as the inhibitory activity against other kinases [28,29]. Our findings reveal that CA induces the polyubiquitination of AXL, thereby reducing AXL levels by promoting its proteasomal degradation. However, AXL may not simultaneously inhibit other kinases with similar catalytic domains (such as c-MET and MERTK kinases) as competitive ATP-binding inhibitors. 

From our results of MTT assay showed that the proliferation of rat astrocyte CTX-TNA2 was moderately decreased to 84.7% ± 5.3% of control in response to 48 h-CA treatment at 20 μM. Our flow cytometry analysis indicated that the cell cycle distribution of the astrocyte was not altered by the same treatment (data not shown). In addition, previous studies also report that CA has several protective effects, including that CA can protect cardiomyocytes from doxorubin-induced cytotoxicity [30], prevent oxidative stress and reduce inflammation [31], and ameliorate non-alcoholic steatohepatitis [32] and diabetes [33]. Therefore, we suggest that CA may not have a cytotoxic effect on astrocytes or at least, only have slight cytotoxicity to astrocytes. Accordingly, we suggest that CA could be a potential treatment for human brain tumors.

AXL and its ligand, GAS6, have been implicated in metastasis and tumorigenesis of various cancers. Recently, GAS6/AXL-triggered actin remodeling has been demonstrated to play an important role in driving the invasion and macropinocytosis of glioblastoma cells in a PI3K-dependent manner [34]. In addition to the PI3K/Akt cascade, GAS6-induced AXL activation and triggers kinase signaling, including ERK and PEAK1, which contribute to the high invasiveness of breast cancer cells [35]. Furthermore, JAK2-activating mutation has been observed in chronic myeloproliferative neoplasms (MPNs), such as chronic myeloid leukemia (CML), polycythemia vera and myelofibrosis [36,37]. However, JAK2 inhibitors have limited clinical success in treating MPNs. It has been demonstrated that AXL is associated with CML resistance, and its inhibitory effect has therapeutic potential in BCR/ABL-resistant CML [38]. Moreover, Pearson et al. reported that inhibiting AXL may be a new therapeutic target for JAK2-induced MPNs [39]. CA induced the glioblastoma cell apoptosis through inhibition of STAT3 and NF-κB activation and induction of apoptotic-related caspases pathways. In addition, CA also reduced tumor proliferation by inhibition of M2 macrophage polarization [18]. However, our results show that GAS6 treatment promotes the p-JAK2, p-ERK and F-actin expression in M059K cells by CA-treated M059K cells. This indicates that GAS6 induces AXL activation and the downstream signaling JAK2/MEK/ERK-dependent F-actin expression. Notably, the GAS6-evoked JAK2/ERK signaling and consequent F-actin polymerization can be diminished by CA, which may result from the downregulation of GAS6 and AXL in response to the direct interaction of CA/GAS6 and CA/AXL as proposed by molecular docking analysis (Figure 7).

Glioma stem cells (GSC) are one of the first types of cancer stem cells isolated from solid tumors, and only 100 GSCs could produce tumors that recapitulate the parental tumors when transplanted into xenograft immunodeficient mice [40]. Two subtypes of GSCs, namely mesenchymal and proneural GSC, have been identified basing on transcriptomic signatures [41]. Notably, AXL is demonstrated as a key regulator for mesenchymal GSC, and knockdown of AXL significantly diminishes the in vitro self-renewal of mesenchymal GSCs and suppresses the in vivo growth of glioblastoma in xenograft mice [13]. In addition to GAS6, it is shown that tumor-associated microglia produce protein S which subsequently interacts with and activates AXL in mesenchymal GSCs and promotes growth of GBM cells, and inhibition of AXL suppresses the promoted growth of GBM cells [42]. Our findings reveal that CA downregulates AXL expression and inhibits AXL-driven signaling, suggesting that CA may have inhibitory effect on mesenchymal GSCs and mesenchymal GSC-promoting GBM growth. However, further investigation is needed. Therefore, our findings indicate that CA can inhibit the migration and invasion of GBM cells and reduce F-actin expression and its polymerization. Additionally, the CA-inhibited invasiveness of GBM cells is attributed to the upregulation of CHIP and subsequent down-regulation of AXL by ubiquitin-mediated proteasome degradation, downregulation of GAS6 and subsequent inhibition of the JAK2/MEK/ERK axis. 

## 5. Conclusions

Thus, these findings reveal that CA has potent anti-metastatic potential against GBM cells and highlight the potential of targeting the AXL/CHIP/GAS6 axis for GBM treatment.

## Data Availability

All experiment data generated or analyzed during in this published article.
